# Attosecond Pulses from a Solid Driven by a Synthesized Two-Color Field at Megahertz Repetition Rate

**DOI:** 10.1021/acsphotonics.5c00410

**Published:** 2025-04-25

**Authors:** Zhaopin Chen, Mark Levit, Yuval Kern, Basabendra Roy, Adi Goldner, Michael Krüger

**Affiliations:** † Department of Physics, 153960 Technion—Israel Institute of Technology, Haifa 32000, Israel; ‡ Solid State Institute, 26747 Technion—Israel Institute of Technology, Haifa 32000, Israel; § The Helen Diller Quantum Center, Technion—Israel Institute of Technology, Haifa 32000, Israel

**Keywords:** attosecond pulses, high-harmonic generation in condensed matter, extreme nonlinear optics, subcycle optical field synthesis, semiconductor Bloch equations

## Abstract

Probing coherent quantum dynamics in light–matter interactions at the microscopic level requires high-repetition-rate isolated attosecond pulses (IAPs) in pump–probe experiments. To date, the generation of IAPs has been mainly limited to the kilohertz regime. In this work, we experimentally achieve attosecond control of extreme-ultraviolet (XUV) high harmonics in the wide-bandgap dielectric MgO, driven by a synthesized field of two femtosecond pulses at 800 and 2000 nm with relative phase stability. The resulting quasi-continuous harmonic plateau with ∼9 eV spectral width centered around 16.5 eV photon energy can be tuned by the two-color phase and supports the generation of an IAP (∼700 attoseconds), confirmed by numerical simulations based on the three-band semiconductor Bloch equations. Leveraging the high-repetition-rate driver laser, the moderate intensity requirements of solid-state high-harmonic generation, and band-structure-induced spectral enhancement, we achieve IAP production at an unprecedented megahertz repetition rate, paving the way for compact all-solid-state XUV sources for IAP generation.

## Graphical abstract

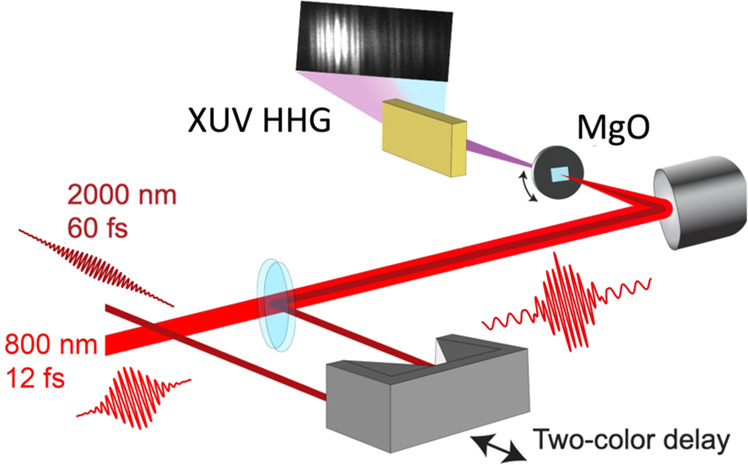

## Introduction

High-harmonic generation (HHG) produces coherent extreme ultraviolet (XUV) light through the interaction of strong laser pulses with gases,^1,2^ solids,^3,4^ and liquids^5^ on a table-top platform. In the time domain, isolated attosecond pulses (IAPs) can be achieved, which are key to the extremely precise pump–probe measurements of attosecond science.^6–8^ Starting with the first successful observation of an IAP in a gas in 2001,^9^ various techniques for producing IAPs from atomic gases have been established,^10^ mostly based on spectral filtering in conjunction with an approach to effectively reduce the number of HHG-generating half-cycles to a single one,^11^ such as polarization gating,^12^ double optical gating,^13^ ionization gating,^14^ and two-color mixing.^15,16^ Another frontier in HHG is the generation of attosecond pulses (APs) at high repetition rates of 100 kHz and higher, which is beneficial for experiments where only low single-shot signal rates can be achieved, such as photoemission spectroscopy and imaging,^17^ or for precision spectroscopy with frequency combs in the XUV.^18^ High repetition rates are essential for improving measurement efficiency, minimizing space charge effects, preventing sample damage, and enhancing the overall signal quality. An early study produced IAPs in a gas at a repetition rate of 600 kHz,^19^ which is, to the best of our knowledge, the highest repetition rate for IAPs.

In principle, HHG from solids^3,20,21^ opens up the possibility to generate IAPs more efficiently than in gaseous media due to the higher atomic density in solids^22^ and the nonparabolic dispersion of the band structure, which enables strong enhancement in certain spectral regions.^23^ The first demonstration of an IAP from a solid has been achieved with synthesized subcycle light field transients generated from a hollow-core fiber supercontinuum at kHz repetition rates.^24,25^ Bulk precompression in a solid enables such transients, opening the door to attosecond pulses.^26^ Solid-state HHG sources in the XUV allow for a more modest peak intensity of the driving laser pulses, on the order of 10^13^ W cm^–2^ or less,^24,26,27^ due to the fact that the band gaps of materials like ZnO, MgO, and SiO_2_ are smaller than the usual ionization energies of noble gases. These properties make solid-state HHG particularly attractive for high repetition rates, alleviating the requirements for the driving laser system. Solid-state HHG has been achieved at tens of MHz oscillator repetition rates using a titanium–sapphire laser in a metal–sapphire nanostructure^28^ and using a mid-infrared (MIR) laser in bulk ZnO.^29^ A first pump–probe spectroscopy application of attosecond pulses generated in ZnO has recently been reported at 100 kHz repetition rate.^30^ IAP generation from solids, however, has not yet been realized at MHz repetition rates, marking an unexplored frontier in attosecond science.

In this work, we employ a laser field synthesized from two incommensurate frequencies and demonstrate high-contrast IAP generation from a wide-bandgap dielectric at a repetition rate of 1 MHz. Compared to commensurate wavelengths,^31,32^ using two incommensurate wavelengths naturally breaks the periodicity of HHG, enabling IAP generation without requiring complex gating mechanisms. Unlike previous incommensurate HHG experiments,^33,34^ the two constituent fields at 800 and 2000 nm wavelength are phase-stable with respect to each other and form the synthesized laser field with controllable waveform on the attosecond scale. We employ a magnesium oxide (MgO) crystal for HHG due to its wide bandgap, high cutoff energy, and spectral enhancements.^23,27,35,36^ These properties facilitate the generation of IAPs. Moreover, MgO exhibits a comparatively high damage threshold, thus allowing us to access the extreme ultraviolet (XUV) spectral range. Through attosecond waveform control, we observe an HHG spectrum with greatly reduced oscillatory features. Our measurement is in excellent agreement with numerical simulations. While we do not directly measure the temporal profile of the generated XUV light field, the excellent agreement between our experimental data and numerical calculations indicates that a high-contrast IAP with a duration of ∼700 as at a center photon energy of 16.5 eV is produced.

## Results

### Synthesized Two-Color Field for High-Harmonic Generation

In our experiment (see Figure 1(a) for a sketch of the setup), we generate high harmonics and implement attosecond control by synthesizing two-color laser pulses using a phase-stable optical parametric chirped pulse amplification (OPCPA) system operating at a repetition rate of 1 MHz (Class 5 Photonics White Dwarf^37^). The OPCPA system outputs two distinct pulses: 12 fs pulses at a wavelength of 800 nm and 60 fs pulses at 2000 nm. Mathematically, the synthesized two-color field can be expressed as *E*(*t*) = *A*_1_ exp­(−2 ln 2 *t* ^2^/τ_1_^2^) cos (ω_1_ *t* + ϕ_CEP,1_) + *A*_2_ exp­(−2 ln 2 (*t* + Δ*t*)^2^/τ_2_^2^) cos (ω_2_ *t* + ϕ + ϕ_CEP,2_), where the subscripts 1 and 2 denote the 800 and 2000 nm laser pulses, and Δ*t* and ϕ are the two-color delay time and phase (ϕ = ω_2_Δ*t*), respectively. The carrier envelope phases (CEPs) ϕ_CEP, 1_ and ϕ_CEP, 2_ are passively stabilized at an unknown fixed value, with a rms stability of 250 mrad over 1 h.^37^ We assume the CEPs to be zero for both colors since they barely affect our results (see the Supporting Information and Figures S4 and S5 for a discussion of the CEP effects). We emphasize the fact that in our case, the two wavelengths are incommensurate, meaning they cannot be related by an integer factor. In our experiment, the pulse energy of the 800 nm beam is maintained at approximately 0.11 μJ, corresponding to a peak power of 8.7 MW after the reflection losses. With a focal spot size of ∼6 μm (1/e^2^ radius), the peak intensity of the 800 nm pump beam is estimated to be approximately 15 TW/cm^2^ at the focus. A weak admixture of the second color (2000 nm) is introduced at a relative intensity of 2%. The 2000 nm beam has a pulse energy of 0.093 μJ, a peak power of 1.46 MW, and a focal spot size of ∼18.2 μm. The relative delay between the two colors is controlled by a translation stage with attosecond precision. We experimentally measured the timing stability using a reference path for the two-color pumps, revealing a two-color delay standard deviation of approximately 45 attoseconds over 20 min, ensuring the temporal stability of the generated attosecond pulses. The resulting synthesized pulse waveform at zero two-color delay is depicted in Figure 1(b). A key characteristic of the synthesized field is the presence of a strongly enhanced field peak in the orange shaded area at the pulse center, while the adjacent field peaks of the 800 nm pulse at *t* ≈ 1.33 and *t* ≈ 2.66 fs are suppressed, indicated by the green circles, due to the constructive or destructive interference of the two fields. The intensity ratio of the synthesized field between the central peak and the strongest adjacent side peak, at around ± 4 fs, is about 72%. Even in the case with a CEP for both pump pulses equal to π/2, the intensity ratio 79% is still sufficient for a good contrast (see Figure S4 in Supporting Information: simulation results for CEP equal to π/2). The synthesized pulse is akin to a few-cycle pulse with a dominant half-cycle electric field (see Figure 1(b)), which enables us to produce a quasi-continuous plateau in the HHG spectrum. Due to the extremely nonlinear nature of the process in play, this ratio can already give rise to a dominant attosecond pulse with large intensity contrast to adjacent pulses, thus equivalent to an IAP. An alternative scheme where the 2000 nm pulse intensity is stronger than the 800 nm pulse faces challenges in achieving IAP generation. The 800 nm pulse cannot effectively suppress the other half-cycle field strengths of the 2000 nm pump, particularly with a pulse duration of 60 fs. This results in a reduced contrast between the main and satellite APs.

**Figure 1:**
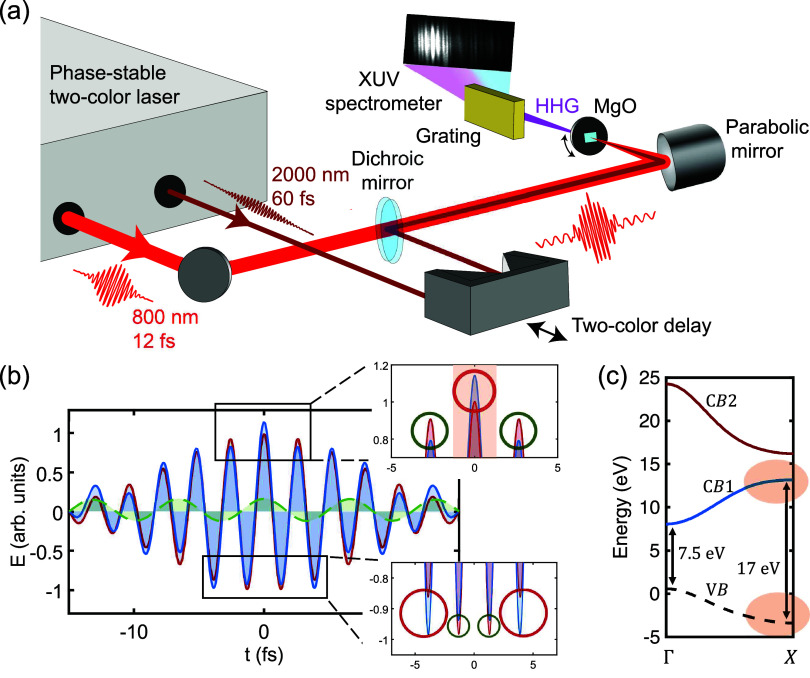
Experimental setup and synthesized two-color field distribution. (a) Experimental setup for attosecond control of high-harmonic generation and isolated attosecond pulse generation from MgO with synthesized two-color light fields. (b) The field profile (solid blue with shading) shows a dominant field peak in the center. Red solid (with shading) and green dashed curves (with shading) depict the 800 and 2000 nm field, respectively. The peak intensity of the 2000 nm field is 2% of the 800 nm peak intensity. The red and green circles in the insets indicate where the 2000 nm field enhances and suppresses the corresponding half-cycle, respectively. (c) Band structure of MgO along the Γ–*X* direction, with the shaded region indicating the spectral range where harmonic yields are enhanced by the Van Hove singularity. VB, CB1, and CB2 denote the valence band, the first conduction band, and the second conduction band, respectively.

We employed an MgO crystal with 50 μm thickness. The crystal is positioned at the focal point of both beams, with the last few hundred nanometers of the sample situated at the center of the beam waist. To optimize the HHG yield, we align the polarization of the laser field along the Γ–*X* orientation of the MgO crystal; see Figure 1(c). This orientation gives rise to a 7.5 eV bandgap at the Γ point and a ∼17 eV bandgap at the *X* point between the valence band and the first conduction band. A strong spectral enhancement is located at the *X* point bandgap,^23^ which we leverage in our experiment. In addition, with sufficiently intense field strength, the second conduction band may also be involved and contribute to higher energy harmonics, up to ∼25 eV.^38^ With this wide bandgap, an XUV spectrum with sufficient spectral width supporting an IAP is feasible. We generate XUV high harmonics in a transmission geometry, with emission occurring within the last ∼10 nm of the crystal—corresponding to the typical attenuation length for this spectral range in MgO^39^—and measure the spectrum using a flat-field XUV spectrometer based on a grating and a microchannel plate (MCP) detector (see Figure 1(a) for a sketch and Section S1 in Supporting Information for further details).

### Attosecond Control of Solid-State High Harmonics

We begin by comparing the high-harmonic (HH) spectra generated by the 800 nm field alone with those induced by the synthesized two-color field, as shown in Figure 2(a,b). The HH spectra driven solely by the 800 nm field exhibit odd harmonic peaks with a spacing of 2*ℏω*_0_ = 3.1 eV, where ω_0_ is the fundamental frequency. Due to the approximately 4.5 cycles contained within the 12 fs duration of the 800 nm pulse, the resulting HHG spectrum shows discrete odd harmonics. At a peak intensity of ∼15 TW cm^–2^, the measured HH spectra exhibit a cutoff energy around 21 eV. Since the MCP detector in our XUV spectrometer is only efficient for photon energies larger than ∼11 eV (see Figure S6 in the Supporting Information: MCP quantum efficiency), our experimental measurement shows this part of the spectrum.

**Figure 2:**
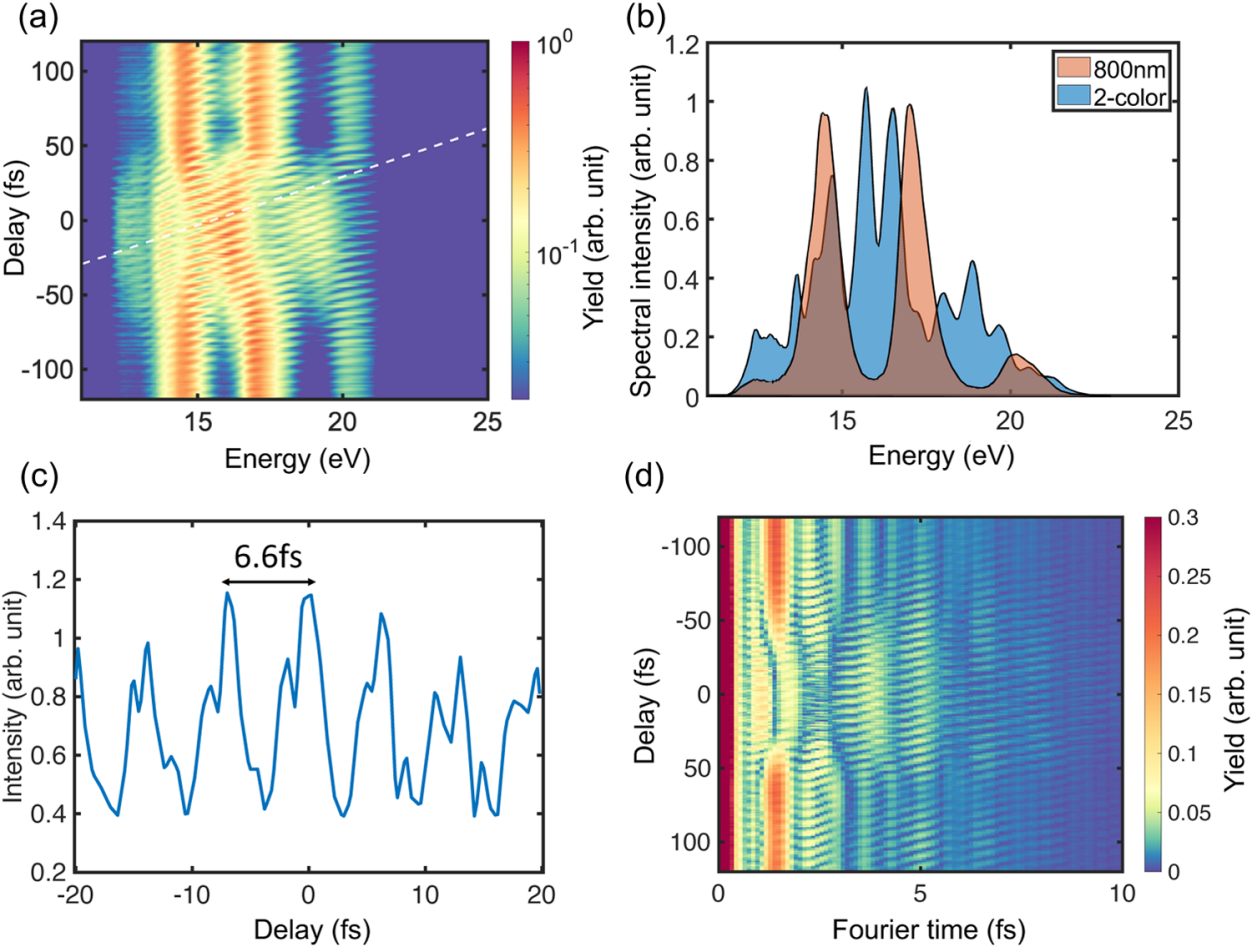
Attosecond-controlled harmonic spectra. (a) Experimentally measured harmonic intensity as a function of two-color delay from –120 to 120 fs. The white dashed line has a slope of 6.5 fs eV^–1^. (b) Experimentally measured harmonic intensity for the 800 nm pulse pump (orange) and for the synthesized two-color field (blue) with 0.5 fs delay. (c) Spectral intensity at 15.5 eV as a function of two-color delay shows a period of 6.6 fs. (d) Harmonic intensity in the Fourier time domain as a function of delay, corresponding to (a).

Introducing the weak 2000 nm laser pulse disrupts the symmetry of the original field, resulting in the occurrence of a dominant peak field at a specific delay. It thus leads to a nearly continuous spectrum with a spectral width of ∼9 eV around the spectral singularity at ∼17 eV. The dominant XUV spectrum is located near the band edge (*X* point), where the Van Hove singularity enhances the HHG yield^23^ (see also the time–frequency analysis in Figure S3 in the Supporting Information). The Van Hove singularity, arising from the high electron density of states at the band edge, is a unique feature of solid-state systems that is absent in gas-phase HHG. Figure 2(a) shows the measured HH spectra as a function of the two-color delay, which is the central result of our work. When the relative delay of two pulses is larger than half of the pulse duration of the 2000 nm, discrete harmonic spectra are mainly produced by the 800 nm pulse. In contrast, the temporal overlapping of the two pulses within a time delay from −30 to 30 fs leads to a quasi-continuous spectrum, with several lower contrast peaks with a spacing of ∼1 eV (see also Figure 2(b)). This already indicates the spectral interference originating from a dominant attosecond pulse and weak subordinate pulses at a temporal spacing of ∼4 fs.

Scanning the delay between the 800 and 2000 nm fields reveals a periodic modulation in the HHG spectrum, as shown in Figure 2(a). The spectral interference forms a tilted stripe-like pattern with a slope of approximately 6.5 fs eV^–1^. A cross-section of the delay-dependent spectral intensity at 15.5 eV in Figure 2(c) highlights a periodic modulation with a temporal period of 6.6 fs. This modulation arises from the temporal distribution of the synthesized two-color field. For incommensurate continuous light waves, the synthesized field can be expressed as *E*(*t*, ϕ) = *A*_1_cos (ω_1_ *t*) + *A*_2_cos (ω_2_ *t* + ϕ), where the field naturally repeats after delays corresponding to the optical periods of each of the two colors. However, for an ultrashort pump pulse consisting of only a few cycles, this repetition behavior no longer holds. The 12 fs duration of the 800 nm pulse is too short to approximate as a continuous wave. On the other hand, the 60 fs 2000 nm pulse can be reasonably treated as a plane wave within the temporal overlap region. As a result, the constructive overlap of the two fields’ peaks occurs periodically, repeating only after one optical cycle of the 2000 nm pulse (see Figure 3(e)).

**Figure 3:**
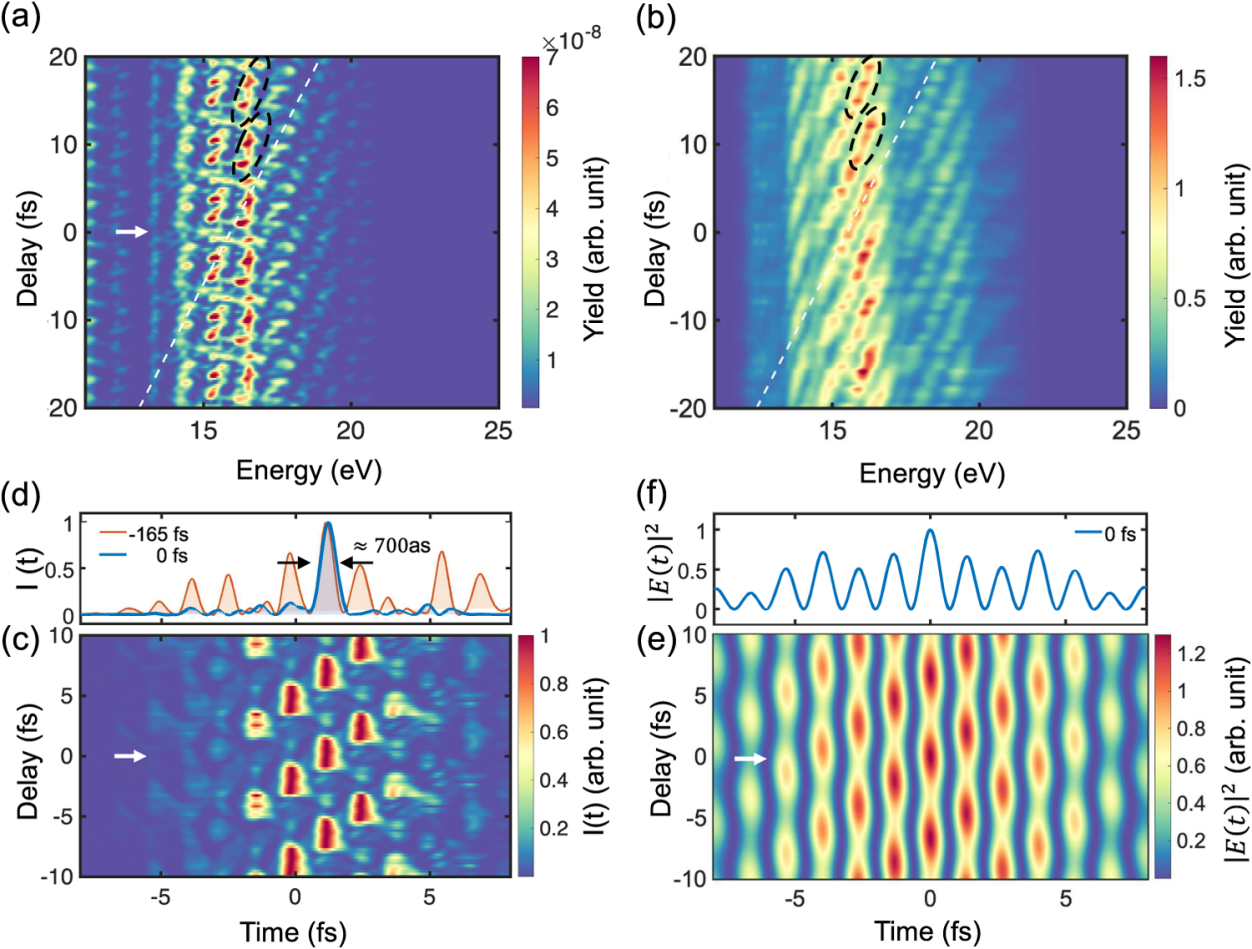
Comparison of experimental results with SBEs simulation results. (a, b) Harmonic intensity as a function of two-color delay for SBE simulation and experiment (logarithmic scale), respectively. For both cases, the peak intensity for the 800 nm pulse is 15 TW cm^–2^. The black dashed circle curves show three local maxima for each 6.6 fs period for both experiment and simulation. The white dashed line for both cases has a slope of 6.5 fs eV^–1^. The white arrow in (a) indicates zero delay. (c) Attosecond pulse intensity as a function of two-color delay in real time. The white arrow indicates zero delay where a high-contrast IAP is produced. (d) Comparison of the formation of attosecond pulse trains and IAPs at time delay −165 fs (orange) and 0 fs (blue), respectively. (e) Field strength square distribution |*E*(*t*)|^2^ of the synthesized pulse as a function of the two-color delay. The white arrow indicates zero delay. (f) The synthetic field strength square distribution with a two-color delay equal to 0 fs.

To better understand the change in harmonic spectra from the non-overlap to the overlap region of our two-color field, we perform an inverse Fourier transformation of the spectral intensity into the Fourier time domain, assuming a uniformly zero spectral phase (see Figure 2(d)). Although the Fourier time domain does not give us access to the actual spectral phase and the exact temporal structure of each attosecond pulse produced in each laser shot, it allows us to extract other crucial information about the train structure of the attosecond pulses. All attosecond pulses contributing to the HHG spectrum and their timing cause interference in the spectral domain and the Fourier transformation can reveal temporal spacings between the attosecond pulses as they are modulated using the two-color delay, in analogy to a HHG spectral interferometry scheme utilizing the CEP.^40^ Out of temporal overlap, the Fourier amplitude plot of the harmonic spectra shows a secondary peak at a Fourier time of τ ∼ 1.3 fs. This peak corresponds to temporal features separated by half a cycle of the 800 nm pulse, showing that a train of attosecond pulses is generated and rendering IAP generation elusive. Remarkably, this changes completely in the overlap region. The secondary peak at 1.3 fs is suppressed due to the enhancement of the electric field in the dominant half-cycle and the suppression of the electric field in the adjacent half-cycles (see Figure 1(b) for a comparison of the electric fields of the 800 nm pulses and the two-color pulses). Additionally, in the overlap region, we find an enhancement of the peak around τ ∼ 4 fs, which corresponds to a temporal delay of attosecond pulses of three optical half-cycles of the 800 nm field. This is the result of constructive interference between the dominant attosecond pulse generated around the peak of the field and one or more weak satellite pulses at a temporal distance of 4 fs.

### Comparison with Numerical Simulations

To elucidate our experimental findings, we utilize the semiconductor Bloch equations (SBEs) to predict and calculate solid-state HHG. The interaction of the synthesized two-color field and the MgO crystal in the Γ–*X* orientation is described by the three-band semiconductor Bloch equations (see Section S1 in Supporting Information for more details). Generally, both interband polarization and intraband current acceleration contribute to HHG in solids. In our case, the HHG yield in MgO is mainly contributed by the interband polarization according to our numerical calculations (see Figure S7 in Supporting Information for a comparison of harmonic spectra arising from intraband and interband contributions), in accordance with prior works.^23,36^ The interband polarization mechanism in solid-state HHG is a three-step recollision process similar to HHG from gases, suggesting the feasibility of generating IAPs in MgO. However, solid-state HHG possesses unique characteristics stemming from the crystal’s band structure, which sets it apart from atomic HHG. This is illustrated in Figures S11 and S12 of the Supporting Information, which compare the harmonic spectra and classical trajectories for the atomic-like and real-band cases in MgO. The band structure not only governs the dynamics of interband transitions but also has the potential to enhance spectral yields, providing opportunities to tailor and optimize the harmonic generation process in solids.

The numerical simulations of the SBEs in Figure 3(a) perfectly match the experimental results in Figure 3(b). Here, the two-color delay is taken from −20 to 20 fs in the overlap region between two pump pulses. Similar to the experimental results, our numerical simulation also displays a 6.6 fs periodicity in the spectral interference as a function of time delay. A tilted stripe-like spectral interference pattern shows a slope of ∼6.5 fs eV^–1^, exactly corresponding to the experimental one. Since the harmonic peak shifts linearly from one order to the next within one cycle of the 2000 nm field, the slope can be expressed as *T*_2μm_/Δ*E* = 6.5 fs eV^–1^, where *T*_2μm_ is the duration of an optical cycle of the 2000 nm field and Δ*E* is the period of the spectral interference from the generated attosecond pulses. Therefore, we can obtain the mean spectral period as Δ*E* ∼ 1.0 eV, which already indicates the interference is mainly from two attosecond pulses with a temporal distance of ∼4 fs, corresponding to 1.5 cycles of the 800 nm field. According to established theory of gas-phase and solid-state HHG,^40–42^ the phase of attosecond pulses, governed by the harmonic dipole phase, depends on the field strength and the time spent by the electron and hole in the conduction and valence bands, respectively. Since the two-color delay periodically changes the field strength in each half-cycle (see the squared absolute value of the synthesized field distribution in Figure 3(e)), the relative harmonic phases contributed by attosecond pulses in different half-cycles vary correspondingly. As a consequence, the spectral interference also shifts as the relative harmonic phases from different attosecond pulses change.

Taking a closer look at the delay dependence of the spectra, a surprising result is that there are fine oscillatory structures within each 6.6 fs period, showing three local maxima of the harmonic intensity within that period for both the simulation and experiment (Figure 3(a,b)). These local maxima originate from the spectral interference of attosecond pulses, indicating the existence of more than one significant attosecond pulse at a specific two-color delay. To verify this assumption, we further use the numerical results to estimate the real-time distribution of the attosecond pulses in Figure 3(c). Here, we can see that the attosecond pulses in real time show a periodic distribution and repeat for each 6.6 fs period of the two-color delay. Within each period, there are three dominant pulses at different two-color delays, along with weak subordinate pulses. The local maxima observed in the spectrum originate from the interference between two dominant attosecond pulses located at the “switch” positions. Since the generation of attosecond pulses is strongly related to the field strength within each half-cycle, we expect that the synthesized two-color field also has a similar temporal distribution. Indeed, the synthesized field strength square distribution |*E*(*t*)|^2^ in Figure 3(e) contains three local maximum values located at (ϕ, *t*) = (0, 0), (π – *πω*_2_/ω_1_, π/ω_1_) and (π + *πω*_2_/ω_1_, – π/ω_1_) for the two-color delay within one cycle of the second color. These electric field local maxima give rise to comparatively more continuous spectra located between the local spectral maxima. Compared with the synthesized field distribution, the half-cycle delay of the attosecond pulse generation indicates that electrons and holes in MgO generated at the original half-cycle will recollide with each other and emit harmonics at the next half-cycle. Although the simulations use idealized electric fields with the measured pulse durations, this approach is valid since the high-harmonic spectra primarily originate from the central cycles, where the field is strongest. Simulations with actual measured electric fields closely align with both experimental results and those using idealized fields (see Figure S13 in the Supporting Information). Further comparison between experiment and simulation regarding the large range of two-color delay in both the spectral and Fourier time domain can be found in the Supporting Information.

## Discussion

The good match between the numerical and experimental results further validates our theoretical model. Therefore, we can use the numerical results to estimate the real-time distribution of the attosecond pulses. For the experimental realization of an IAP, a filter that acts as a spectral window to remove the fundamental light and lower-order harmonics is required. Here, we consider an indium filter,^43^ which has a transparency window between 12 and 21 eV photon energy. To compare the attosecond pulse distribution generated by one- and two-color fields, we examine the intensity distribution of the generated attosecond pulse at two specific delay positions (see Figure 3(d)). At a delay time of 0 fs, an IAP with a full width at half-maximum (FWHM) duration of approximately 700 as is generated under the synthesized field shown in Figure 3(f), as obtained from the numerical solution of SBEs. The timing of IAP in this case remains nearly unaffected by variations in the two-color delay within a temporal window of approximately 1.4 fs. Without the indium filter, the attosecond pulse exhibits a longer duration (approximately 800 as) and more pronounced satellite pulses (see Figure S17 in the Supporting Information for a comparison of attosecond pulse distributions without and with the indium filter). At a delay of −165 fs, where two pump pulses are widely separated, an attosecond pulse train is generated due to the discrete high-harmonic spectra. By controlling the two-color delay, we can switch between an IAP and a pulse train and use their corresponding spectral signatures as a guide.

It is important to note that we extracted the IAP temporal profile from the spectral amplitude and phase calculated from the SBEs. However, direct extraction of the spectral phase from SBEs may introduce artifacts and lead to unreliable values.^44^ In our case, we find that the IAP duration calculated with the help of the numerical spectral phase from the SBEs agrees reasonably well with that of another approach. To illustrate this more clearly, we extract the classical emission time by identifying the time corresponding to the maximum harmonic intensity using a time–frequency (Gabor) analysis of the simulated harmonic spectra (see Figure S14 in the Supporting Information). Figure 4 presents the spectral intensity and group delay distribution of the simulated high-harmonic spectrum, obtained under the same conditions as those in Figure 3(d). We further calculate the atto-chirp parameter^45^ as *C* = d*t*/d­(*ℏω*) = 55 as/eV, corresponding to a group delay dispersion (GDD) ≈ *ℏC* = 0.036 fs^2^. It should be emphasized that the atto-chirp parameter captures only the second-order dispersion, while higher-order dispersion terms in the spectral phase can also influence the pulse duration. Due to the Van Hove singularity enhancement near the bandgap, which dominates the spectral contribution in Figure 4(a) and the short main driver laser wavelength of 800 nm, the attosecond chirp remains small. The spectral phase can be obtained by integrating the emission time *t*_e_. Compared to a transform-limited pulse with a duration of 580 as, the reconstructed pulse based on the classical emission times has a duration of approximately 600 as (see Figure 4(b)), in reasonable agreement with the result shown in Figure 3(d).

**Figure 4:**
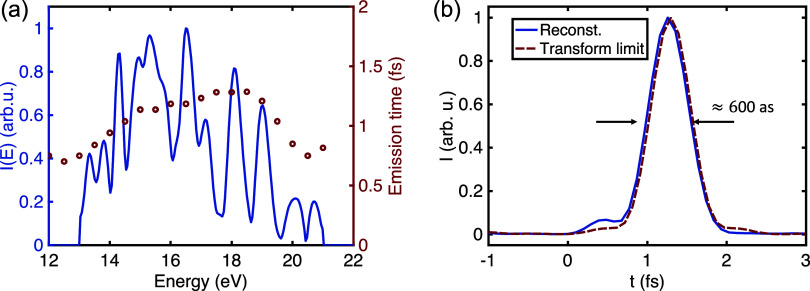
(a) Spectral intensity and emission time distribution of the simulated high-harmonic spectrum, obtained under the same conditions as in Figure 3(d) The emission times are obtained from a time–frequency analysis of the simulation results. (b) The corresponding temporal intensity profile, with a pulse duration of approximately 600 as (blue solid curve), obtained via Fourier transform of the spectral intensity and emission times shown in (a) For comparison, the red dashed-dotted curve represents the transform-limited pulse with a duration of 580 as.

It is also essential to discuss the optimal parameters of the bichromatic fields for IAP generation in MgO. To establish the conditions for IAP generation, we define a criterion where the maximum intensity ratio between the satellite pulses and the main attosecond pulse is ϵ ≤ 0.2. Systematic analysis using semiconductor Bloch equation simulations shows that for a second-color pump wavelength of 2 μm, an intensity ratio of at least 0.12% relative to the 800 nm pump peak intensity is required for IAP generation. Furthermore, with the second-color peak intensity fixed at 2% of the 800 nm pump, our analysis indicates that the optimal wavelength for satisfying the ϵ = 0.2 criterion is approximately 1.46 μm or longer. Further details regarding the optimal conditions of the two-color fields are provided in Section S9 and Figures S8–S10 of the Supporting Information.

In conclusion, we demonstrate attosecond control of HHG from the dielectric MgO, driven by a combination of two femtosecond pulses with incommensurate wavelengths and a controllable relative phase. By precisely controlling the temporal overlap of the two-color field, we synthesize the equivalent of a single-cycle pulse, producing a quasi-continuous XUV spectrum without the need for complex single-cycle femtosecond pulse compression techniques. This result is confirmed by our theoretical model based on the semiconductor Bloch equations, showing the creation of an IAP. By combining the advantages of a two-color optical parametric chirped pulse amplification system, featuring high repetition rates, phase stability, and ultrashort pulse durations, alongside the moderate intensity requirements of solid-state HHG and band structure-induced spectral enhancement, we achieve IAP generation at 1 MHz repetition rate. With photon energies in the lower XUV range (∼10 to ∼20 eV), our high-repetition-rate IAPs are ideally suited for pump–probe experiments, enabling the study of attosecond electron dynamics in wide-bandgap dielectrics such as LiF,^46^ deep valence-to-conduction band excitations in solid materials,^47^ and molecular charge migration.^48^

In the future, direct measurement of the IAP generated in our setup by attosecond streaking will be of significant interest. For instance, part of the two-color beam could be split off and recombined with the generated VUV-EUV attosecond pulses by using a holey mirror to facilitate streaking measurements. By employing frequency-resolved optical gating for the complete reconstruction of attosecond bursts (FROG-CRAB,^12,49^ ), the temporal profile of the attosecond pulses can be reconstructed. Here, the main experimental challenge lies in selecting an appropriate atomic gas with an ionization energy matching the photon energy of the generated VUV-EUV pulses, which is in the range of 13–21 eV. For example, alkali atoms are suitable candidates (cf.^30^).

## Supplementary Material



## Data Availability

Data underlying the results presented in this paper are not publicly available at this time but may be obtained from the authors upon reasonable request.

## Notes

Semiconductor Bloch equations (SBEs) for numerical simulation; details of the experimental setup; band structure and transition dipole moment of MgO; SBEs simulation results for CEP equal to zero; SBEs simulation results for CEP equal to π/2; quantum efficiency of the microchannel plate detector; comparison of harmonic spectrum from intra- and interband currents; optimal intensity and wavelength of second-color pump for IAP generation in MgO; enhancement of harmonic yields by Van Hove singularity; classical trajectories between atomic and solid HHG; simulation results with full temporal pulse profiles reconstructed from FROG; group delay and chirp of high-harmonic pulse; emission time of classical motion equation; influence of CEP phase fluctuations on IAP; attosecond pulse generation with and without an indium filter (PDF)

## Notes

Z.C., M.L., Y.K., B.R., A.G. and M.K., Attosecond pulses from a solid driven by a synthesized two-color field at megahertz repetition rate, 2024, arXiv:2410.09178. URL: https://arxiv.org/abs/2309.00734, (accessed Sep 18, 2024).
