# Supplementary material for: Attosecond Pulses from a Solid Driven by a Synthesized Two-Color Field at Megahertz Repetition Rate
Source: ACS Photonics. 2025 Apr 25;12(5):2819–27. doi: 10.1021/acsphotonics.5c00410 (PMC12100772; doi:10.1021/acsphotonics.5c00410)
Supplement: Supplementary file 1 [file ph5c00410_si_001.pdf]

Supporting Information: Attosecond pulses from a solid driven by a synthesized two-color field at megahertz repetition rate

Zhaopin Chen,[1,2,3,*] Mark Levit,[1,2,3,*] Yuval Kern,[1,2,3]
Basabendra Roy,[1,2,3] Adi Goldner,[1,2,3] and Michael Krüger[1,2,3]

[1]*Department of Physics, Technion—Israel Institute of Technology, Haifa 32000, Israel*
[2]*Solid State Institute, Technion—Israel Institute of Technology, Haifa 32000, Israel*
[3]*The Helen Diller Quantum Center,*
Technion—Israel Institute of Technology, Haifa 32000, Israel

SUPPLEMENTARY TEXT

1. Semiconductor Bloch equations calculations

To describe the interaction of the synthesized two-color field and the MgO crystal in the Γ-X orientation, we use the following one-dimensional, three-band semiconductor Bloch equations (SBEs):

$$
\begin{aligned}
i\frac{\partial}{\partial t}P_k^{\nu c_1}(t) &= \left[\epsilon_k^{c_1} - \epsilon_k^{v} - i\frac{1}{T_2} + iE(t)\nabla_k\right] P_k^{\nu c_1}(t) - \left(1 - f_k^{c_1}(t) - f_k^{v}(t)\right) d_k^{c_1\nu} E(t) \\
&\quad + E(t)\left(d_k^{c_2\nu} P_k^{c_2 c_1}(t) - d_k^{c_1 c_2} P_k^{\nu c_2}(t)\right) \\
i\frac{\partial}{\partial t}P_k^{\nu c_2}(t) &= \left[\epsilon_k^{c_2} - \epsilon_k^{v} - i\frac{1}{T_2} + iE(t)\nabla_k\right] P_k^{\nu c_2}(t) - \left(1 - f_k^{c_2}(t) - f_k^{v}(t)\right) d_k^{c_2\nu} E(t) \\
&\quad + E(t)\left(d_k^{c_1\nu} P_k^{c_1 c_2}(t) - d_k^{c_2 c_1} P_k^{\nu c_1}(t)\right) \\
i\frac{\partial}{\partial t}P_k^{c_1 c_2}(t) &= \left[\epsilon_k^{c_2} - \epsilon_k^{c_1} - i\frac{1}{T_2} + iE(t)\nabla_k\right] P_k^{c_1 c_2}(t) + \left(f_k^{c_2}(t) - f_k^{c_1}(t)\right) d_k^{c_2 c_1} E(t) \\
&\quad + E(t)\left(d_k^{\nu c_1} P_k^{\nu c_2}(t) - d_k^{c_2\nu}\left(P_k^{\nu c_1}(t)\right)^*\right)
\end{aligned}
\tag{S1}
$$

$$
\begin{aligned}
\frac{\partial}{\partial t}f_k^{\nu}(t) &= -2\mathrm{Im}\left[d_k^{c_1 v}E(t)\left(P_k^{\nu c_1}(t)\right)^* + d_k^{c_2 v}E(t)\left(P_k^{\nu c_2}(t)\right)^*\right] + E(t)\nabla_k f_k^{\nu}(t) \\
\frac{\partial}{\partial t}f_k^{c_1}(t) &= -2\mathrm{Im}\left[d_k^{c_1\nu}E(t)\left(P_k^{\nu c_1}(t)\right)^* + d_k^{c_1 c_2}E(t)\left(P_k^{c_2 c_1}(t)\right)^*\right] + E(t)\nabla_k f_k^{c_1}(t) \\
\frac{\partial}{\partial t}f_k^{c_2}(t) &= -2\mathrm{Im}\left[d_k^{c_2 v}E(t)\left(P_k^{\nu c_2}(t)\right)^* + d_k^{c_2 c_1}E(t)\left(P_k^{c_1 c_2}(t)\right)^*\right] + E(t)\nabla_k f_k^{c_2}(t)
\end{aligned}
\tag{S2}
$$

Here, $P_k^{\lambda,\lambda'}(t)$ represents a dimensionless polarization depending on time t and momentum k, induced by the time-dependent electric field $E(t)$, with $\lambda, \lambda' = \{v, c_1, c_2\}$. The indices v, c_1, and c_2 correspond to the valence band, first conduction band, and second conduction band, respectively. $f_k^{\lambda}(t)$ denotes the population of electrons or holes in the conduction or valence bands. The energy-momentum dispersion relation for the band ϵ_k^{λ}, as well as the transition dipole moments (TDMs) $d_k^{\lambda\lambda'}$, are derived from density functional theory (DFT) calculations[1]. The exact band structure and TDMs used in our calculations are plotted in Fig. S1. Here, we consider a "smooth-periodic" gauge such that TDMs maintain smoothness and periodicity at the boundary of the k-space, preserving the crystal symmetry.

---

* These authors contributed equally to this work.

This ensures the absence of unphysical even-order harmonics in a single-color field from an inversion-symmetric crystal structure like MgO. Since MgO is an inversion-symmetric crystal, the transition dipole phase (TDP) is trivial[2] and does not influence the evaluation of the generated attosecond pulse. We set the phenomenological decoherence time T_2 to half of the optical cycle duration of the 800 nm laser. This approach cuts off long electron-hole trajectories and achieves good agreement with the experiment.

The HHG spectrum is computed as

$$S_{\mathrm{HHG}}(\omega) \sim \left| \int_{-\infty}^{\infty} [J_{\mathrm{inter}}(t) + iJ_{\mathrm{intra}}(t)] e^{i\omega t} dt \right|^2 , \tag{S3}$$

where

$$J_{\mathrm{inter}}(t) = \sum_{\lambda,\lambda'} \frac{d}{dt} \int d_k^{\lambda\lambda'} \cdot (P_k^{\lambda\lambda'}(t))^* dk + \mathrm{c.c.} \tag{S4}$$

and

$$J_{\mathrm{intra}}(t) = \sum_{\lambda} \int \nu_k^{\lambda} f_k^{\lambda}(t) dk \tag{S5}$$

denote the interband and intraband contributions to the HHG yield, respectively. $\nu_k^\lambda = \nabla_k \epsilon_k^\lambda$ represents the group velocity of the electrons and holes in the corresponding bands.

2. Details of the experimental setup

The two-color delay is controlled by a closed-loop linear piezo stage (Physik Instrumente P-611.1). For combining the two colors, we use a dichroic mirror (Thorlabs DMLP1180T). Starting with the focusing optics, the HHG setup is situated in an ultra-high vacuum chamber with a base pressure of $\sim 1 \times 10^{-8}$ mbar. The laser beam is focused using an off-axis parabolic mirror with a focal length of 5 cm. The spot size of the 800 nm beam at the focus is $\sim$6 μm 1/e^2 radius, as measured by a camera. The 2000 nm beam at the focus has an effective radius of $\sim$18.2 μm, as determined by a knife-edge measurement. The pulse durations were measured using frequency-resolved optical gating. The $< 100 >$-cut MgO crystal used in our experiment has a thickness of 50 μm (Farview Optics). The home-built spectrometer uses a 100 μm wide entrance slit and a flat-field diffraction grating suitable for wavelengths of $50 - 200$ nm (L0120-50-200, Shimadzu).

We also determine the divergence angle of the generated harmonics by recording the $1/e^2$ width at the MCP position and the distance between the MCP detector and the MgO

crystal. H11 has a divergence angle of approximately 17.9 mrad. With a pump intensity of 15 TW cm^{-2}, we find that the lower bound of the photon flux of the measured HHG signal is on the order of 10^7 photons per second in the spectral range from 10 eV to 20 eV. For this estimate, we take into account the grating and the low detection efficiency of our uncoated MCP (Texel MCP-50-D-S-P43).

In our OPCPA system[3], a diode-pumped femtosecond industrial laser with a central wavelength of 1030 nm, a pulse energy of 60 µJ, and a repetition rate of 1 MHz is used as the primary pump source. A fraction of the 1030 nm beam is split off and directed to drive white-light continuum generation (WLG), which seeds a difference frequency generation (DFG) stage. In this stage, the 1030 nm fundamental combines with the white-light seed to generate a passively carrier-envelope-phase (CEP) stable idler at 2 µm with a rms stability of ~ 250 mrad (~ 150 mrad with active stabilization).

The 2 µm idler is subsequently amplified through a two-pass non-collinear optical parametric amplification (NOPA) process. For the 800 nm output, a second NOPA stage is employed, where the seed is derived from a second white-light continuum (WLG2) driven by a fraction of the CEP-stable 2 µm output. As a result, the CEP stability of the 2 µm pump is directly transferred to both the 800 nm output and the second white-light continuum.

In this manner, the pulses at both 800 nm and 2 µm are passively phase-stable, with active control further suppressing the CEP noise.

3. Band structure and transition dipole moment of MgO for numerical calculations

The three-band structure and transition dipole moment (TDM) of MgO along the $\Gamma - X$ orientation in our numerical simulation is obtained from a density functional theory calculation[1]. We also introduce a small modification of the TDM amplitude to better fit the experimental results. We show the band structure and TDM in Fig. S1.

4. Semiconductor Bloch equation simulation results for CEP equal to zero

Here, we present additional simulation results of the two-color control of high harmonic generation from MgO, based on the semiconductor Bloch equations (1) and (2), using the same parameters as those in Fig. 3 of the main text. Figure S2(a) displays the harmonic

spectrum as a function of the two-color delay, ranging from $-120\,\mathrm{fs}$ to $120\,\mathrm{fs}$. Similar to the experimental results in Fig. 2(a) in the main text, the spectrum shows a quasi-continuous profile in the central overlap region of the two-color pulses. Spectral interference shifts as a function of delay are also clearly observed in the simulation. The white dashed line indicates a slope of $6.5\,\mathrm{fs\,eV^{-1}}$, consistent with the experimental observations. In the Fourier time domain, Fig. S2(b) reveals a suppression of the secondary peak at a Fourier time of $t \approx 1.33\,\mathrm{fs}$ and the appearance of weak intensity at $t \approx 4\,\mathrm{fs}$, closely resembling the experimental data in Fig. 2(d) of the main text.

In order to see the harmonic distribution in both time and frequency domain for a specific delay, we perform a Gabor transformation over the interband and intraband currents obtained by Eqs. (3) and (4) in the main text. The Gabor transformation is calculated by

$$G(\omega, t') = \left| \int \left[J_{\mathrm{inter}}(t) + J_{\mathrm{intra}}(t)\right] \exp\left(-\frac{(t-t')^2}{\sigma_t^2}\right) e^{i\omega t}\, dt \right|^2 . \tag{S6}$$

Here, we use a Gaussian window function with a width of $\sigma_t = 800\,\mathrm{as}$. Figure S3(a) clearly shows the generation of an isolated pulse with a spectrum ranging from $13\,\mathrm{eV}$ to $20\,\mathrm{eV}$ at a two-color delay time of $\tau = 0\,\mathrm{fs}$. At $\tau = 2.5\,\mathrm{fs}$, two attosecond pulses with a temporal separation of $4\,\mathrm{fs}$ are produced. It is evident that the attochirp is minimal in both cases, primarily due to the contribution of the short trajectory.

5. Semiconductor Bloch equation simulation results for CEP equal to $\pi/2$

In order to assess the influence of CEP of both pulses on the experimental results, we consider a case with $\phi_{\mathrm{CEP},1} = \phi_{\mathrm{CEP},2} = \pi/2$, which is the opposite with respect to Fig. 3 in the main text with zero CEP phase. Even with $\pi/2$ phase for 800 nm where the positive and negative peak intensity are equal in the center of the pulse, a high intensity ratio between the dominant peak and secondary peak is still obtained, $\sim 79\%$ (see Fig. S4(a)). The corresponding attosecond pulse distribution from the generated high harmonics is highly similar to the one with $\phi_{CEP,1} = \phi_{CEP,2} = 0$ in Fig. 3(c) in the main text. In this case, an isolated attosecond pulse with high contrast can still be realized under specific delay, e.g., $\tau = 0.9\,\mathrm{fs}$ (see Fig. S4(b)). The spectral intensity as a function of two-color delay in Fig. S4 is very similar to the case of zero CEP. Our results show that the CEP does not affect the main physics and behaviours in our two-color HHG experiments, except for a small influence

on the IAP duration ($\sim 100\,\mathrm{as}$).

6. Quantum efficiency of the micro-channel plate detector

Figure S6 shows the estimated quantum efficiency of the micro-channel plate (MCP) detector used to measure high harmonics in the experiment. To simulate the experimental results, we also multiplied the SBE simulation spectrum by the MCP efficiency.

7. Comparison of harmonic spectrum from intra- and interband currents

Figure S7 compares the harmonic spectral intensity contributions from intraband and interband currents driven by an 800 nm pulse with a peak intensity of $15\,\mathrm{TW\,cm^{-2}}$. Strikingly, the harmonic intensity generated by the interband current is several orders of magnitude higher than that from the intraband current.

8. Optimal intensity and wavelength of second-color pump for IAP generation in MgO

As shown in Fig. S8(a), the satellite attosecond pulses are significantly suppressed as the 2 µm intensity ratio increases. To define the conditions for IAP generation, we set a criterion where the maximum intensity ratio between the satellite pulses and the main pulse is $\epsilon \leq 0.2$. In Fig. S8(b), we highlight the intensity ratios of the two most prominent satellite pulses relative to the main attosecond pulse. When the 2 µm intensity reaches approximately 0.12% of the 800 nm peak intensity—corresponding to an intensity ratio of 88% between the two largest intensity maxima of the synthesized field—this criterion is met, and IAP generation is achieved. Therefore, for the case of using 2 µm as the second-color pump, an intensity ratio of 0.12% or higher relative to the 800 nm peak intensity is required for IAP generation.

Additionally, we analyzed attosecond pulse generation as a function of the second-color pump's central wavelength. Figure S9 demonstrates that when the second color peak intensity is fixed at 0.12% of the 800 nm pump, the satellite pulses are further suppressed as the wavelength increases, particularly the dominant satellite located at 0.22 fs. Once the

central wavelength reaches 2 μm, the ratio between the satellite and the main attosecond pulse decreases to the critical value of 20% (Fig. S9(b)). This suggests that for wavelengths longer than 2 μm, IAP generation becomes increasingly robust. Moreover, when the peak intensity of the second-color field is increased to 2% of the fundamental, our analysis (Fig. S10) reveals that the optimal wavelength for meeting the $\epsilon = 0.2$ criterion is approximately 1.46 μm or longer.

9. Enhancement of harmonic yields by Van Hove singularity

In the case of IAP generation from MgO crystals, the dominant XUV spectrum is located at the band edge (X point), where the Van Hove singularity enhances the HHG yield[4] (see also the time-frequency analysis in Fig. S3). The Van Hove singularity, arising from the high electron density of states at the band edge, is a unique feature of solid-state systems that is absent in gas-phase HHG. To further illustrate the differences between atomic and solid HHG, we compared high harmonic spectra generated from a real MgO band structure with those from a parabolic band structure (simulating the atomic case) with an effective mass equivalent to MgO at the Γ point.

As shown in Fig. S11, for a fundamental pump intensity of 15 TW/cm^2 and a 2% 2 μm pump intensity, the real MgO band exhibits a Van Hove singularity enhancement between 13 eV and 20 eV near the X-point bandgap. In contrast, the parabolic band structure lacks such enhancement, resulting in significantly lower harmonic yields in the same spectral region. These distinctions highlight the unique role of solid-state band structures in shaping the HHG process.

10. Classical trajectories between atomic and solid HHG

We also analyzed classical trajectories of electron (hole) ionization and recombination based on a semiclassical model and electron (hole) equations of motion. Under identical pump conditions (800 nm central wavelength, field strength $F = 0.0236$ a.u.), the atomic-like MgO system with a parabolic band exhibits clear short and long trajectories (Fig. S12(a)). In the solid case, multiple trajectories are observed, with the dominant ones (highlighted by green shading) corresponding to negative field ionization[5] — an effect unique to solid HHG

(see Fig. S12(b)). When comparing these classical trajectories to the trajectories calculated via Gabor transformation, the dominant classical trajectories align well, highlighting the contributions of valence and conduction bands (see Fig. S12(c)). It is noteworthy that the classical trajectories in the solid case, calculated with only two bands (valence and first conduction), do not account for harmonics exceeding the bandgap energy.

11. Simulation results with full temporal pulse profiles reconstructed from FROG

While the SBE simulations in the main text were performed using ideal electric fields with pulse durations measured by frequency-resolved optical gating (FROG), we also considered the full temporal pulse profiles reconstructed from the FROG measurements. The pulse distributions for both the 800 nm and 2 μm pulses are shown in Fig. S13(a) and (b). The frequency-domain simulation results as a function of two-color delay, shown in Fig. S13(c), closely match both the experimentally measured results and the simulations using ideal electric fields. The isolated attosecond pulse generated in this case has a duration of approximately 650 as, consistent with the result obtained using ideal electric fields in Fig. 3 of the main text and Fig. S4.

These findings validate the use of ideal pulses with measured durations, as the harmonic yields and spectra are primarily determined by the central cycles of the pulses, which are largely unaffected by the envelope shape of the surrounding regions.

12. Group delay and chirp of high-harmonic pulse

As discussed in the main text, our numerical simulations account for both the spectral phase and classical emission time of the high harmonics. Specifically, we solve the semiconductor Bloch equations to obtain the spectral intensity and phase of the harmonics. Classical trajectories are extracted by identifying the emission time corresponding to the maximum harmonic intensity via a Gabor transformation of the simulated spectra, as shown in Fig. S14(a). The group delay dispersion (GDD) of the simulated attosecond pulse is estimated to be GDD = 0.036 fs^2. In Fig. S14(b), we compare the pulse duration of transform-limited case with the chirped case incorporating this GDD. The results show that for a GDD of this magnitude, the shortest achievable pulse duration is approximately

450 as for a 5.8 eV FWHM bandwidth. Therefore, the generation of an isolated attosecond pulse with a duration of $\sim$ 700 as, as observed in our simulations, is consistent with these expectations.

13. Emission time of classical motion equation

In Fig. S15(a), we analyze the classical emission trajectories based on the electron (hole) equations of motion, using the same two-color field conditions as in Fig. 3(d) of the main text. When compared to the trajectories extracted via the Gabor transformation, we find that the dominant classical trajectories (highlighted by a white dashed square) are in good agreement. The corresponding GDD calculated by classical model is GDD $= 0.046$ fs^2,which is close to the value obtained from the Gabor analysis in Fig. 4 of the main text. However, since the classical calculation is limited to a two-band model (valence and first conduction bands), it cannot account for high harmonics that exceed the bandgap energy—specifically, those in the range of approximately 17–21 eV.

To estimate the pulse duration, we calculate the classical emission time and manually extend it to 21 eV, as shown in Fig.S15(b). By combining this emission time (and thus spectral phase) with the harmonic intensity spectrum obtained from the semiconductor Bloch equations (SBEs), we reconstruct an attosecond pulse with a duration of approximately 600 as, as shown in Fig.S15(c).

14. Influence of CEP phase fluctuations on IAP

We compare the isolated attosecond pulse (IAP) generated under two conditions: one with a maximum CEP fluctuation of $\sim$250 mrad and another with zero CEP fluctuation (see Fig. S16). In both cases, an isolated attosecond pulse with a duration of $\sim$700 as is produced, with central time jittering around 150 as.

15. Attosecond pulses generation with and without indium filter

It is relevant to consider the generation of attosecond pulses both with and without the indium filter, as the lower-order harmonics contribute to the generated XUV pulses, influ-

encing the attosecond pulse profile in the absence of the filter. To highlight this effect, we compare the attosecond pulse distribution with and without the indium filter, as shown in Fig. S17. As illustrated in Fig. S17(a), when the indium filter is not used, the attosecond pulse exhibits a longer duration ($\sim$ 800 as) and stronger satellite pulses. This demonstrates the filter's effectiveness in suppressing lower-order harmonics, thereby improving pulse isolation.

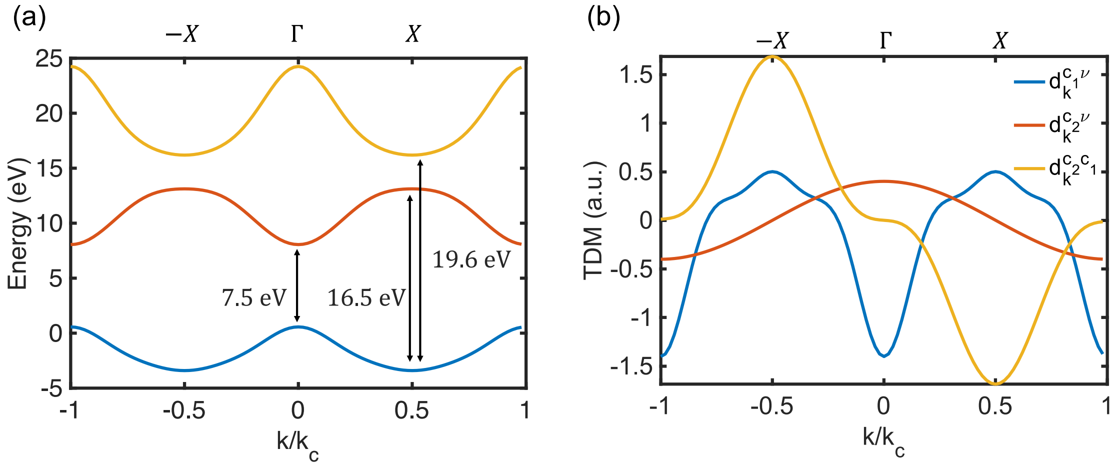

Figure S1. Band structure and transition dipole moment of MgO. (a) Band structure of the MgO crystal in the $\Gamma - X$ orientation. The bottom curve is the valence band and the upper two curves represent the first and second conduction bands, respectively. (b) Corresponding transition dipole moments (TDMs) in atomic units (a.u.). Here, k_c is defined as $k_c = 2\pi/a$, where $a = 7.78$ (atomic unit) is the lattice constant of MgO in the $\Gamma - X$ orientation.

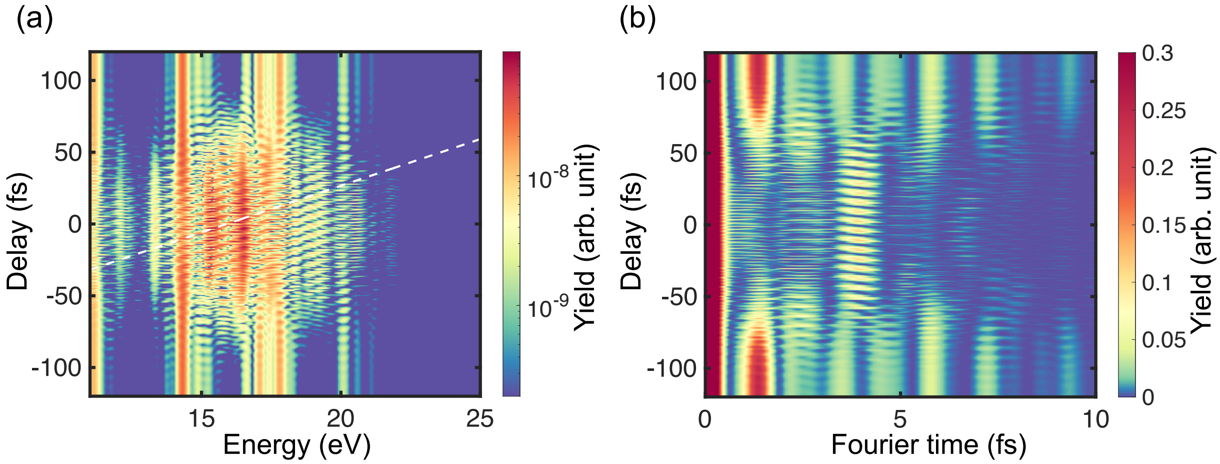

Figure S2. Harmonics intensity in spectral and Fourier time domain. (a) Harmonic spectrum as a function of two-color delay from $-120\,\mathrm{fs}$ to $120\,\mathrm{fs}$ calculated with the SBEs. The white dashed line has a slope of $6.5\,\mathrm{fs\,eV^{-1}}$. (b) Harmonic intensity in the Fourier time domain as a function of delay, corresponding to (a).

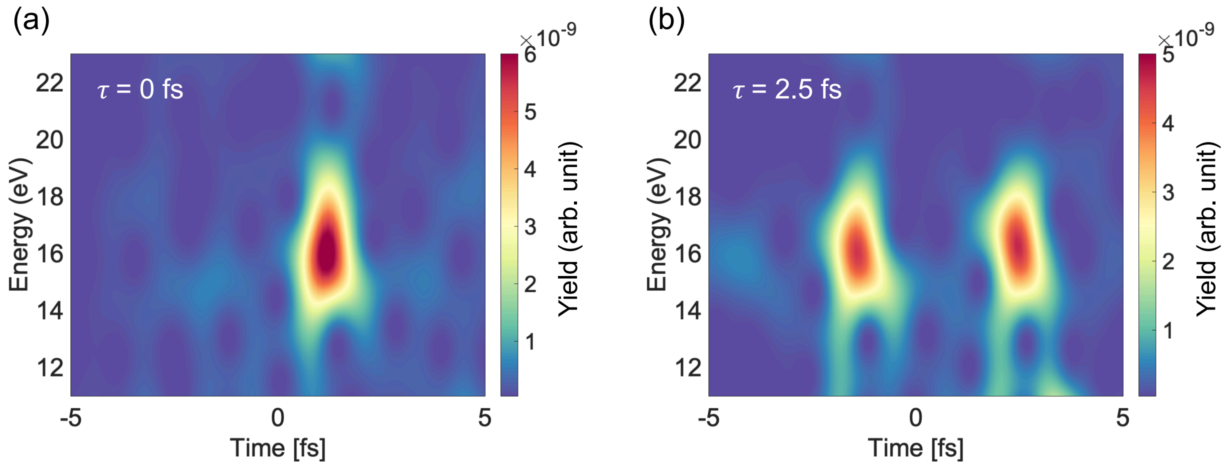

Figure S3. Time-frequency analysis. Gabor transformation of the simulated harmonics in Fig. 3 of the main text, based on the definition in Eq. S6. (a) and (b) correspond to two-color delays $\tau = 0\,\text{fs}$ and $\tau = 2.5\,\text{fs}$, respectively.

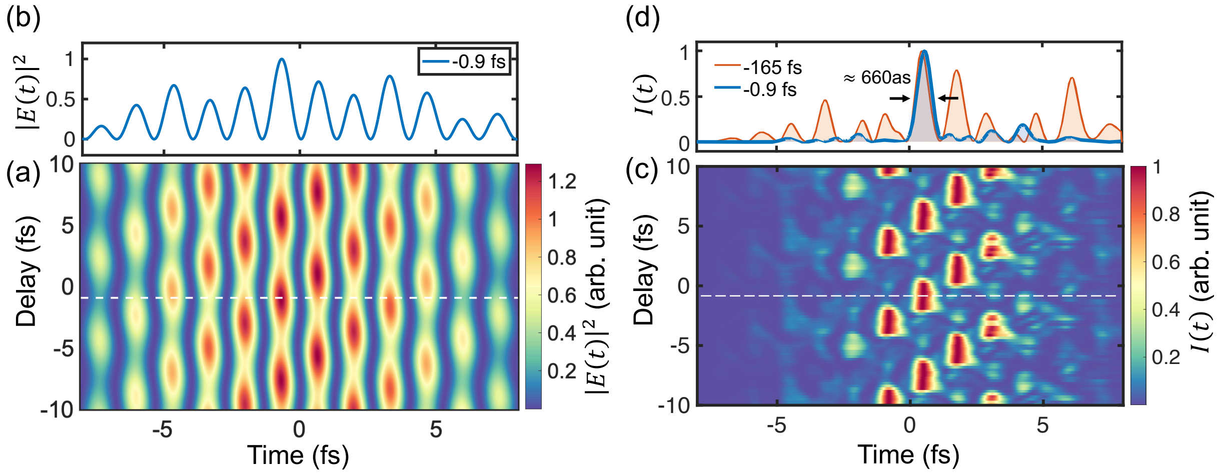

Figure S4. Time-domain simulation results for CEP equal to $\pi/2$. (a) Field strength square $|E(t)|^2$ distribution of the synthesized pulse as a function of the pulse delay. (b) shows the synthetic field distribution in the white dash line with two-color delay equal to -0.9 fs. The intensity ratio between the dominant peak and secondary peak is $\sim 79\%$. (c) Attosecond pulses intensity as a function of two-color delay in real time. (d) compares the formation of attosecond pulse trains and isolated attosecond pulses at time delay -165 fs (orange) and -0.9 fs (blue), respectively. The dash line in the lower plot indicates the corresponding time delay. Similar to Fig. S2, the peak intensity for the 800 nm pulse is $15\,\mathrm{TW\,cm^{-2}}$, but with the CEP $\phi_{\mathrm{CEP}} = \pi/2$.

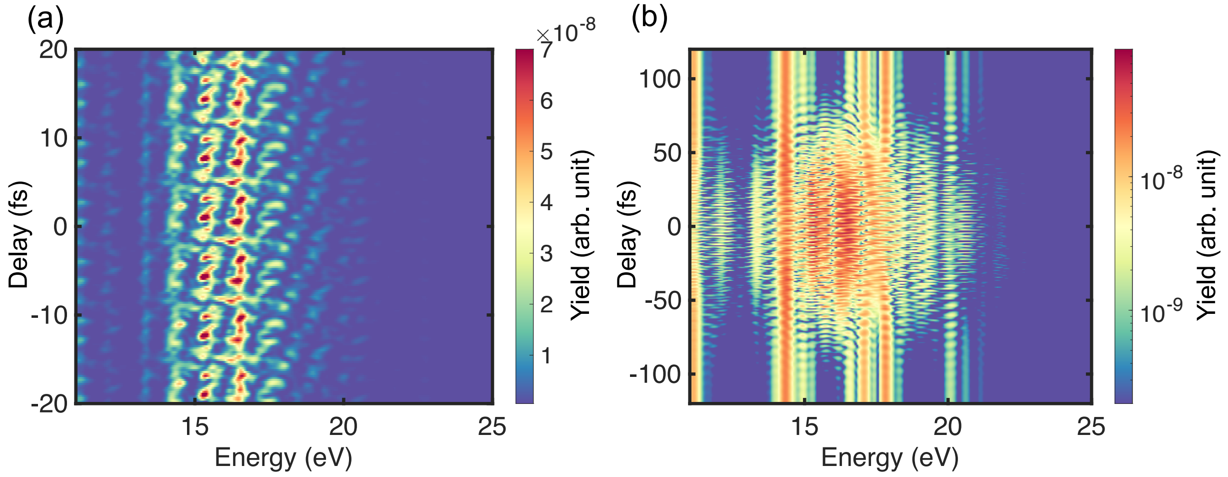

Figure S5. Frequency-domain simulation results for CEP equal to $\pi/2$. Harmonic intensity as a function of two-color delay for SBE simulation for (a) range from -20 fs to 20 fs, and (b) from -120 fs to 120 fs. Similar to Fig. S3, the peak intensity for the 800 nm pulse is $15\,\mathrm{TW\,cm^{-2}}$, but with CEP phase $\phi_{\mathrm{CEP}} = \pi/2$.

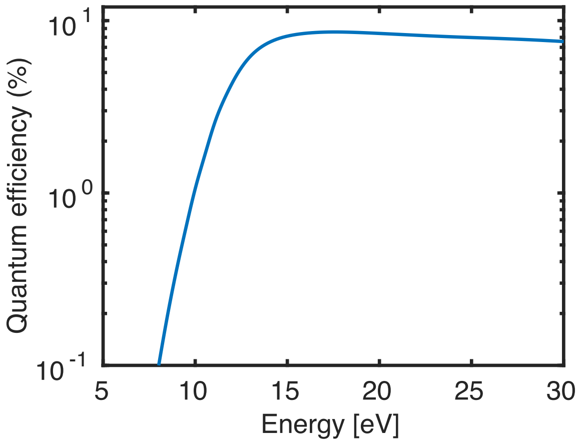

Figure S6. MCP quantum efficiency. Estimated quantum efficiency of the microchannel plate detector as a function of photon energy. For the SBE simulation results in this paper, we account for this efficiency to model the experimental results.

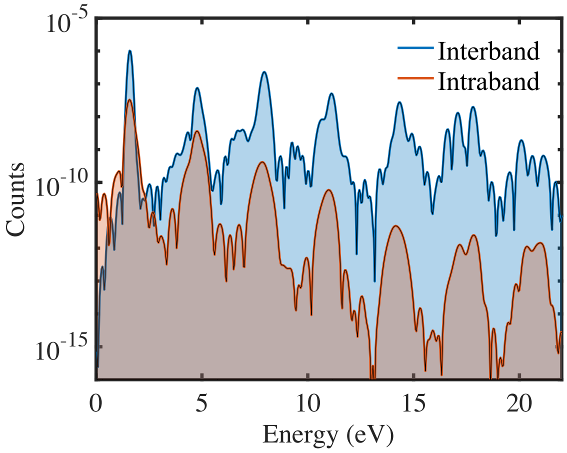

Figure S7. Harmonics spectrum from interband and intraband currents. Comparison between interband and intraband contribution to the harmonic spectrum for a single-color 800 nm pump with $15\mathrm{TW\,cm^{-2}}$.

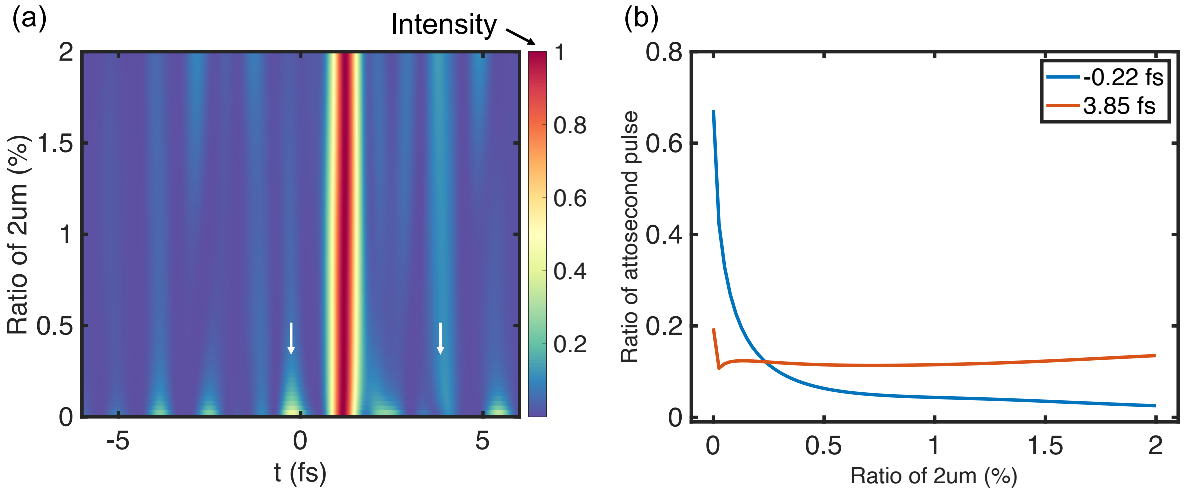

Figure S8. (a) Dependence of attosecond pulse generation on the 2 μm intensity ratio relative to the 800 nm peak intensity. (b) Intensity ratio between the main attosecond pulse and the satellite pulses at delays of -0.22 fs and 3.85 fs, plotted as a function of the 2 μm intensity ratio.

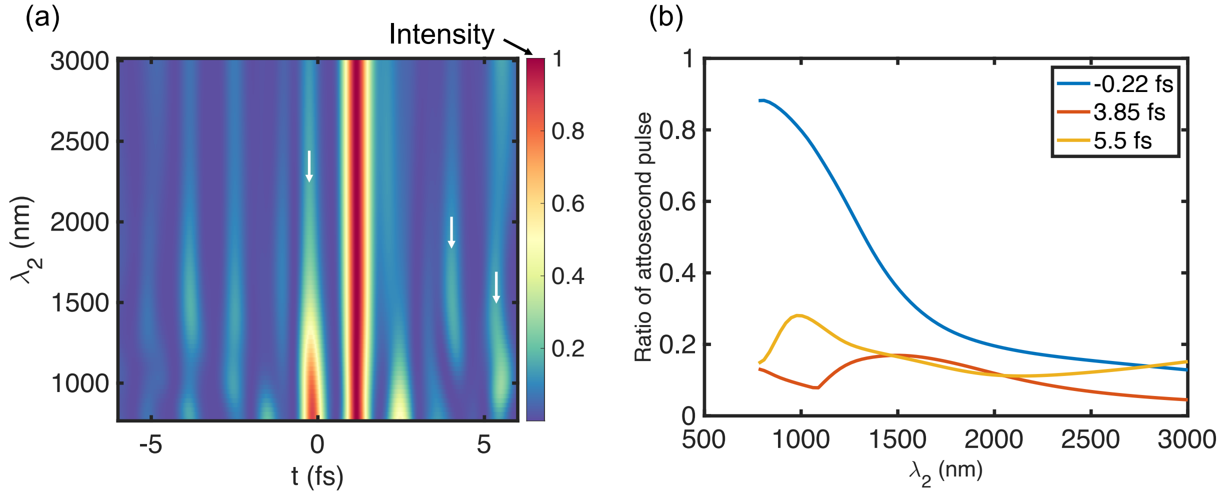

Figure S9. (a)Dependence of attosecond pulse generation on the central wavelength of the second-color pump, with the second-color peak intensity fixed at 0.12% of the 800 nm peak intensity. (b) Intensity ratio between the satellite pulses (at -0.22 fs, 3.85 fs, and 5.5 fs) and the main attosecond pulse, as a function of the central wavelength of the second-color pump.

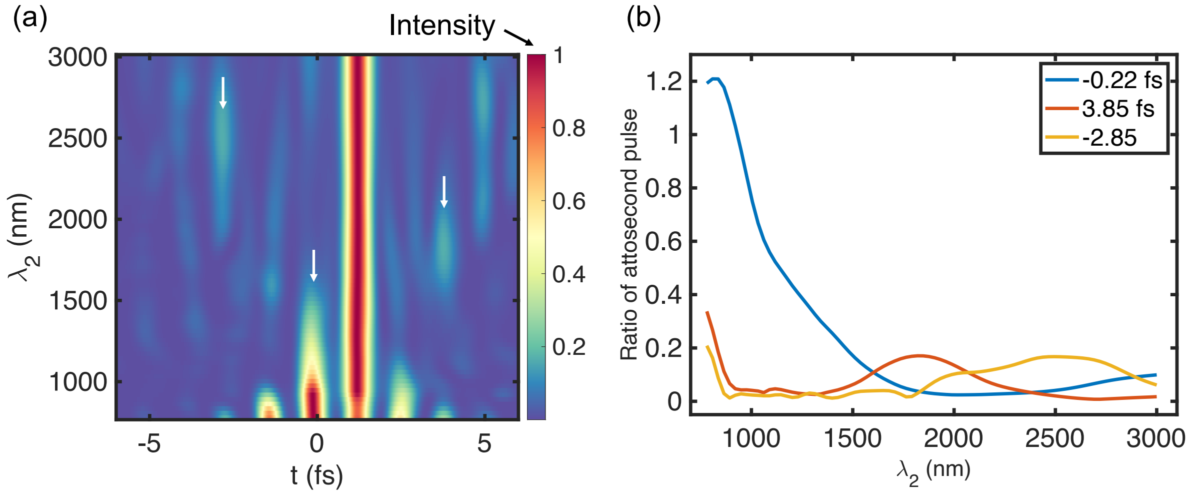

Figure S10. (a) Dependence of attosecond pulse generation on the central wavelength of the second-color pump, with the second-color peak intensity fixed at 2% of the 800 nm peak intensity. (b) Intensity ratio between the satellite pulses (-2.85 fs, -0.22 fs, and 3.8 fs) and the main attosecond pulse, as a function of the central wavelength of the second-color pump.

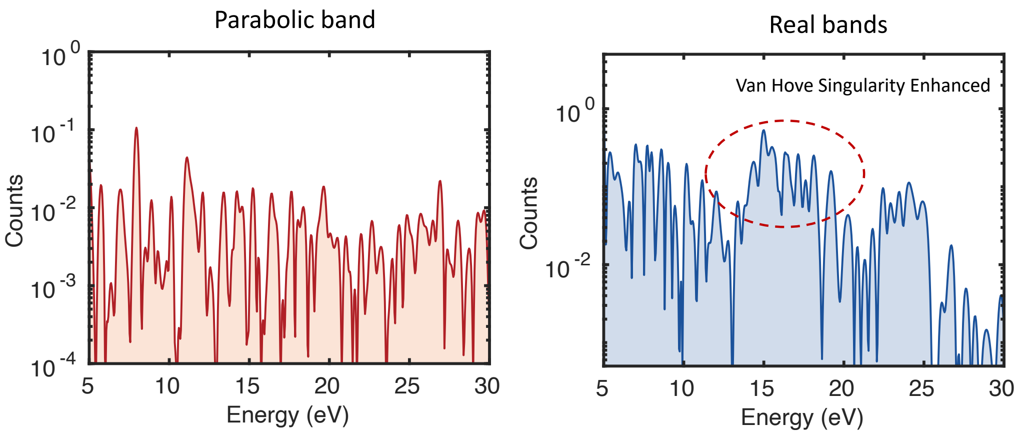

Figure S11. Comparison of high harmonic spectra generated using (a) a parabolic band structure and (b) the real band structure of MgO. The parabolic band, with an effective mass equivalent to that of MgO at the Γ point, is used to simulate the atomic-like case of MgO. The fundamental pump intensity is set to 15 TW/cm^2 with a 2% 2 μm second-color pump. The Van Hove singularity is clearly observed in the 13–20 eV region for the real MgO band structure.

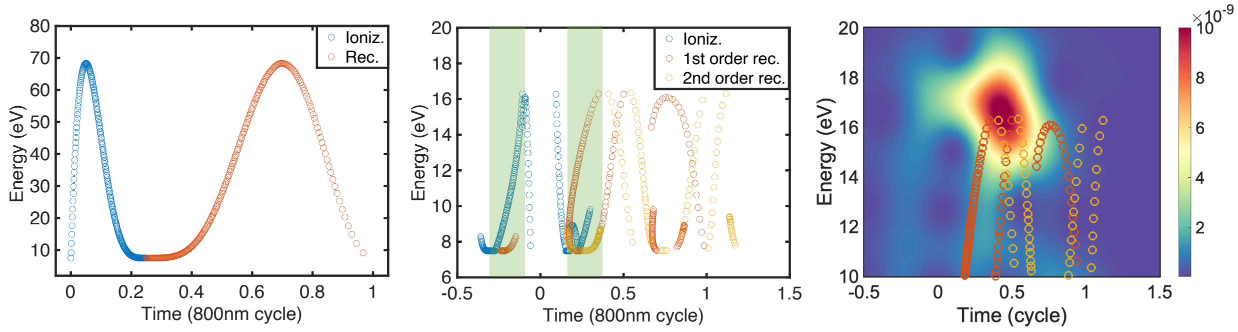

Figure S12. Comparison of classical electron (hole) trajectories based on motion equations for (a) a parabolic band structure and (b) the real band structure of MgO, under a peak field strength of $F = 0.0236$ (atomic units), corresponding to an intensity of $1.95\,\mathrm{TW/cm^2}$. In the atomic-like HHG case (a), distinct short and long trajectories are evident, whereas in the solid-state case (b), multiple trajectories emerge. The dominant trajectory, associated with the highest harmonic intensity, is highlighted by the green shaded area. (c) Time-frequency analysis of HHG in solid MgO, performed using Gabor transformation, under an 800 nm pump pulse with a 4 fs duration and the same field strength as in (a) and (b). The analysis shows that the dominant classical trajectory closely aligns with the quantum trajectory revealed by the Gabor transformation.

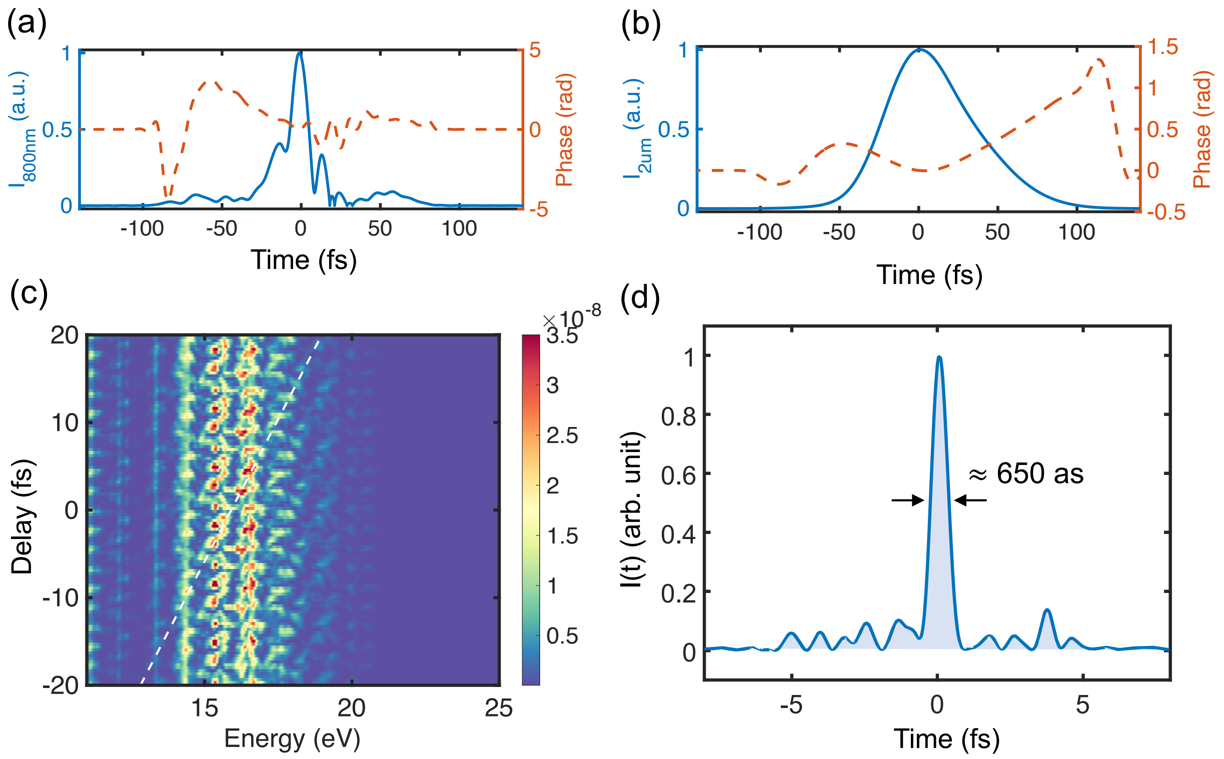

Figure S13. Temporal profiles of the (a) 800 nm and (b) 2 μm pump pulses reconstructed from FROG measurements. The 800 nm pulse has a duration of 12 fs, while the 2 μm pulse has a duration of 60 fs. (c) Frequency-domain simulation results using the FROG-measured electric fields, showing the harmonic intensity as a function of two-color delay over a range from -20 fs to 20 fs. (d) An isolated attosecond pulse with a temporal duration of approximately 650 as is generated at a time delay of 1 fs.

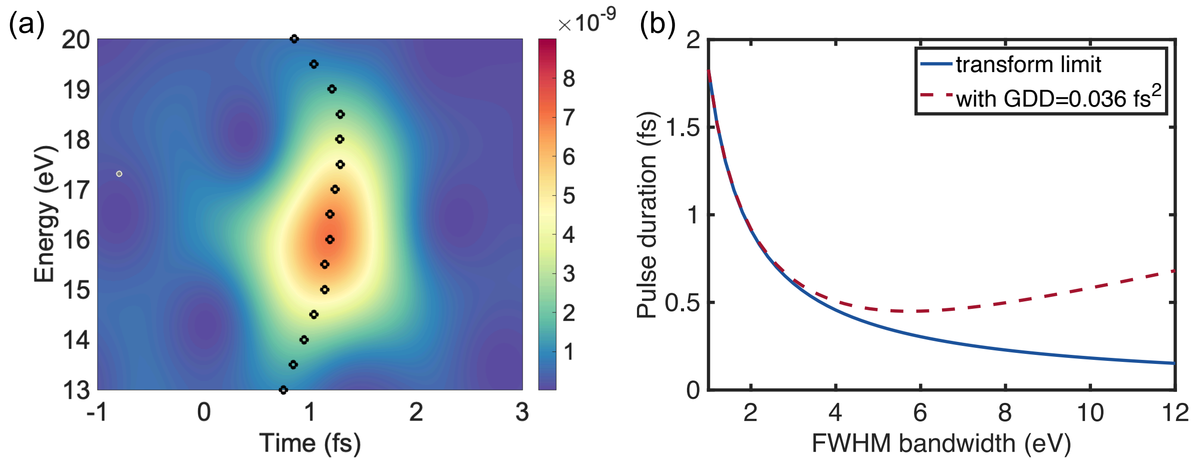

Figure S14. (a) Gabor transformation of the simulated harmonics in Fig. 3 (d) of the main text, based on the definition in Eq. (S6). Here, the two-color delay is $\tau = 0$, the same as shown in Fig. 3(d) of the main text. The classical emission time trajectory is obtained by taking the time corresponding to the maximal harmonic intensity. (b) Comparison of the attosecond pulse in the transform limit and the one with the calculated chirp, GDD ≈ 0.036 fs^2.

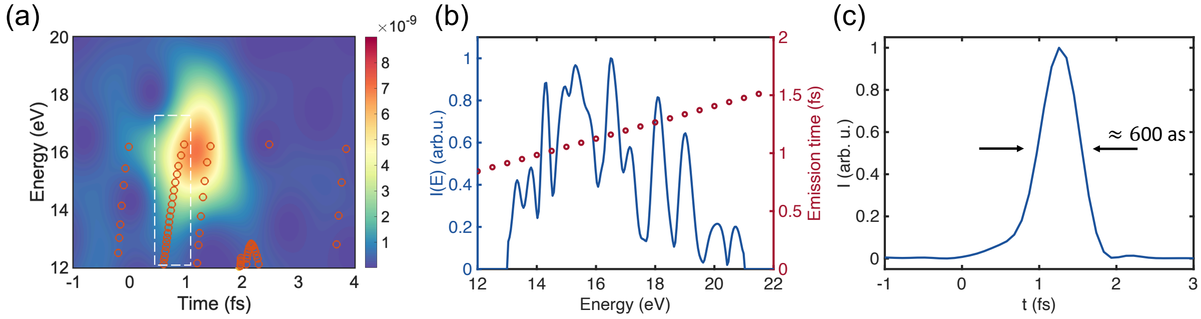

Figure S15. (a) Gabor transformation of the simulated harmonics in Fig. 3 (d) of the main text. Orange circle curve shows the corresponding emission time trajectory. The dominant trajectory, associated with the highest harmonic intensity, is highlighted by dashed white square. The analysis shows that the dominant classical trajectory closely aligns with the quantum trajectory revealed by the Gabor transformation. (b) Extended emission time distribution according to panel (a), and the spectral intensity. (c) The corresponding temporal intensity profile, with a pulse duration of approximately 600 as.

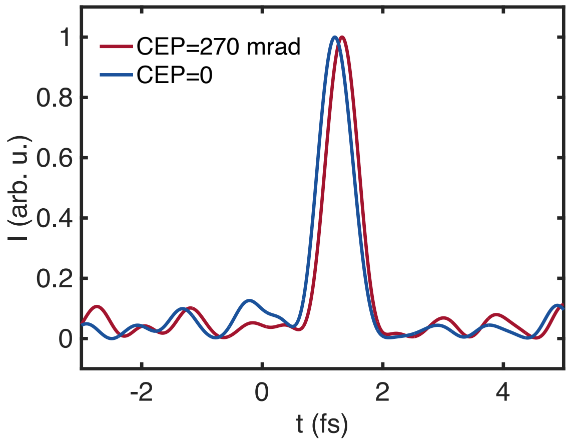

Figure S16. Comparison of isolated attosecond pulse temporal distribution with CEP phase equal to 0 mrad and 270 mrad, which is the maximum CEP noise. The pulse duration for both cases is approximately 700 as, with central time jittering around 150 as. The fundamental pump intensity is set to 15 TW/cm^2 with a 2% 2 μm second-color pump, and the two-color delay is equal to 0 fs.

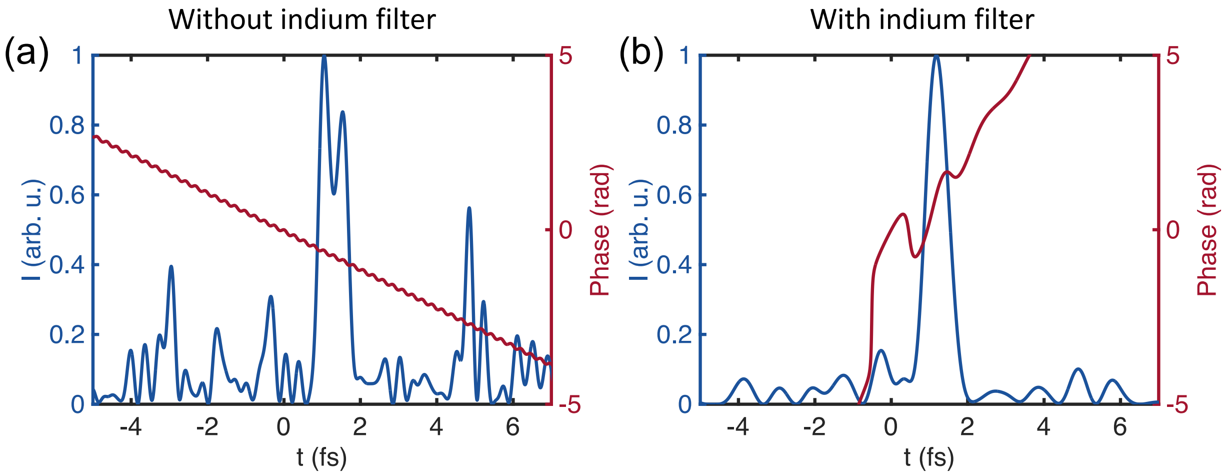

Figure S17. Comparison of attosecond pulse temporal distribution without and with the indium filter. Here, the fundamental pump intensity is set to 15 TW/cm^2 with a 2% 2 μm second-color pump, with the two-color delay equal to 0 fs.

REFERENCES

[1] S. Jiang, C. Yu, J. Chen, Y. Huang, R. Lu, and C. D. Lin, Smooth periodic gauge satisfying crystal symmetry and periodicity to study high-harmonic generation in solids, Phys. Rev. B **102**, 155201 (2020).

[2] S. Jiang, H. Wei, J. Chen, C. Yu, R. Lu, and C. Lin, Effect of transition dipole phase on high-order-harmonic generation in solid materials, Physical Review A **96**, 053850 (2017).

[3] T. Braatz, E. Zapolnova, S. Starosielec, I. Grguraš, T. Golz, M. Prandolini, J. H. Buss, M. Schulz, and R. Riedel, 1 MHz - dual channel, CEP stable, few-cycle OPCPA at 800nm and >1.7µm wavelength, in *Nonlinear Frequency Generation and Conversion: Materials and Devices XX*, Vol. 11670, edited by P. G. Schunemann and K. L. Schepler, International Society for Optics and Photonics (SPIE, 2021) p. 116700Z.

[4] A. J. Uzan, G. Orenstein, Á. Jiménez-Galán, C. McDonald, R. E. F. Silva, B. D. Bruner, N. Klimkin, V. Blanchet, T. Arusi-Parpar, M. Krüger, A. N. Rubtsov, O. Smirnova, M. Ivanov, B. Yan, T. Brabec, and N. Dudovich, Attosecond spectral singularities in solid-state high-harmonic generation, Nat. Photon. **14**, 183 (2020).

[5] G. Vampa, C. R. McDonald, G. Orlando, P. B. Corkum, and T. Brabec, Semiclassical analysis of high harmonic generation in bulk crystals, Phys. Rev. B **91**, 064302 (2015).
